# Pancreaticobiliary maljuction combining with pancreas divisum: Report of four cases

**DOI:** 10.3892/etm.2013.1403

**Published:** 2013-11-12

**Authors:** YIN ZHANG, WENSHENG SUN, FAMING ZHANG, JIN HUANG, ZHINING FAN

**Affiliations:** 1 Department of Digestive Endoscopy and Medical Center for Digestive Diseases, Second Affiliated Hospital of Nanjing Medical University, Nanjing, Jiangsu 210011, P.R. China; 2 Department of Hepatobiliary Surgery, Traffic Hospital of Shandong Province, Jinan, Shandong 250000, P.R. China

**Keywords:** pancreaticobiliary maljunction, pancreas divisum, diagnosis, therapy

## Abstract

Pancreaticobiliary maljunction (PBM) is an unusual anomalous condition in which the pancreatic duct and bile duct merge outside the duodenal wall and form a long common channel. Pancreas divisum (PD) is a congenital anomaly in which the dorsal and ventral pancreatic ducts fail to fuse. Endoscopic retrograde cholangiopancreatography (ERCP) is the gold standard for diagnosing PD and magnetic resonance cholangiopancreatography (MRCP) is the non-invasive choice. In this study, four cases of patients with unusual PBM in addition to PD are described. The patients presented with abdominal pain, which was caused by distal biliary stricture diagnosed by MRCP. The patients received ERCP and had a good prognosis.

## Introduction

Pancreaticobiliary maljunction (PBM) is an unusual congenital anomaly, defined as an anatomical maljunction of the pancreatic duct and the biliary duct outside of the duodenal wall (1). PBM has an increased risk of various complications including cholelithiasis, cholangitis, pancreatitis and biliary tract malignancy (2). According to a Japanese study group, PBM may be classified into three different types in which: i) the common bile duct (CBD) joins the major pancreatic duct (MPD); ii) the MPD joins the CBD; iii) the fuse union was not seen clearly (3). Pancreas divisum (PD) is the most common anatomic variant of the pancreas with dorsal and ventral pancreatic glands draining separately into the duodenum (4).The overall endoscopic detection rate for PD was ~2.9% pooled in a systematic review in 2009, with rates worldwide varying from 2.7 to 22% (5,6). Furthermore, the coexistence of MPD and PD is extremely rare clinically, making the complex situation difficult to diagnose and treat. The present study describes four patients who were successfully diagnosed with PBM combined with PD and subjected to endoscopic treatment. Written informed consent was obtained from each patient.

## Case reports

### Case 1

**Figure 1:**
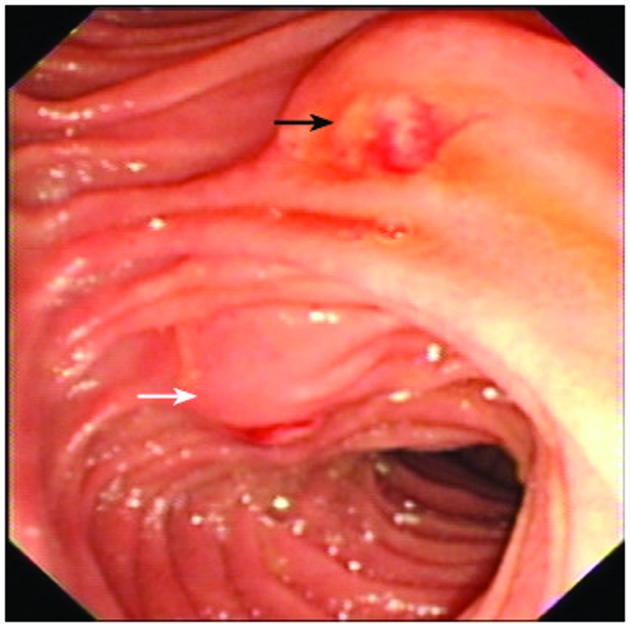
Case 1. Endoscopic image reveals the major papilla (white arrow) and the minor papilla (black arrow) from which bile drained out.

**Figure 2:**
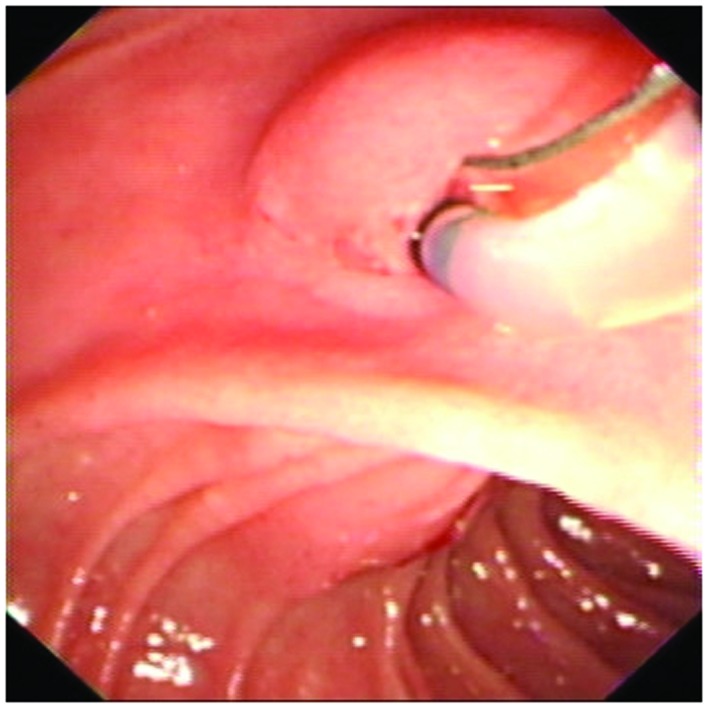
Case 1. Endoscopic image shows the cannulation to the minor papilla.

**Figure 3:**
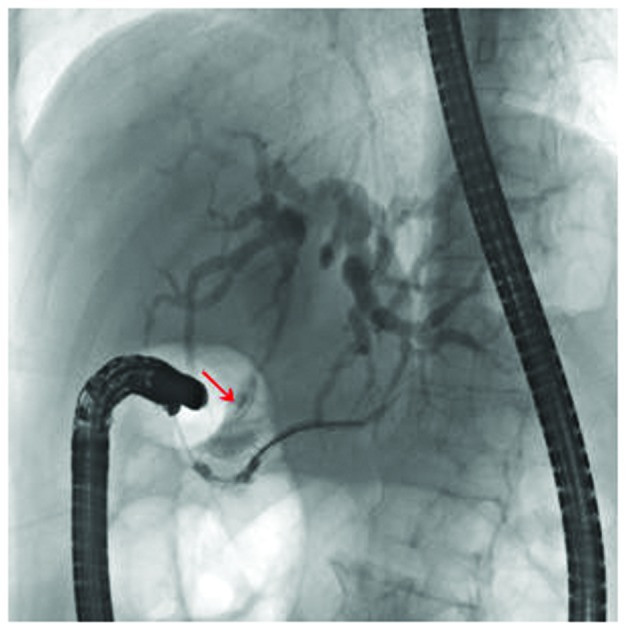
Case 1. Fluoroscopy image shows a stricture (red arrow) at the fusion position of the distal common bile duct (CBD) and pancreatic duct and dilation of the CBD.

An 11-year-old female, complaining of persistent abdominal pain in the right epigastrium with nausea and vomiting for two days, was hospitalized in The Second Affiliated Hospital of Nanjing Medical University (Nanjing, China). Physical examination revealed right upper abdominal tenderness without jaundice. Laboratory tests revealed an elevated white blood cell (WBC) count [WBC, 12.0×10^9^/l; neutrophil (NE), 68.9%] and a high level of transaminases (alanine transaminase (ALT), 532.1 U/l; γ-glutamyl transpeptidase (GGT), 152 U/l), while the serum bilirubin and amylase levels were normal. A computed tomography (CT) scan demonstrated that the intrahepatic bile duct and upper CBD of the patient were dilated and a 10×10mm flake shadow with low density was observed in the distal CBD. No imaging features indicative of pancreatitis were observed. Magnetic resonance cholangiopancreatography (MRCP) confirmed the stricture of the distal CBD. An endoscopic retrograde cholangiopancreatography (ERCP) was performed, but failed on the first attempt due to difficult cannulation. In the secondary procedure one week later, cannulation of the major papilla to biliary duct failed again (Figs. 1 and 2). However, cannulation was successful when the minor papilla was used and X-ray imaging revealed that the guidewire had reached the accessory pancreatic duct. Injection of a contrast agent into the accessory pancreatic duct revealed intra- and extra-hepatic bile duct dilation and distal CBD stricture with its orifice on the accessory pancreatic duct (Fig. 3). After two months, the minor papilla was cannulated to the biliary duct through the accessory pancreatic duct and a pigtail plastic pancreatic stent was implanted into biliary duct following endoscopic sphincterotomy (EST). The patient’s symptoms were resolved and did not return. The stent was removed six months later and the patient remained asymptomatic during the 2 years follow-up.

### Case 2

A 57-year-old female presented to our academic center (Second Affiliated Hospital of Nanjing Medical University) after experiencing 17 days of paroxysmal upper abdominal cramps and a fever. Physical examination revealed jaundice, subcostal and right upper abdominal tenderness and hepatic percussive pain. Routine blood tests revealed normal hematological results, while the liver biochemistry function test suggested cholestasis (ALT, 201 U/l; aspartate transaminase (AST), 90 U/l; total bilirubin (TBIL), 77 μmol/l; indirect bilirubin (IBIL), 51.1 μmol/l; alkaline phosphatase (ALP), 251 U/l; GGT, 198 U/l). The levels of amylase were normal. B-mode ultrasound revealed cystic dilation of the average-upper CBD, partial intrahepatic bile ducts and choledocholithiasis. MRCP revealed dilation of the upper CBD (25 mm in diameter) with a rat-tailed stricture in the lower CBD. The first ERCP failed due to difficult cannulation from the major papilla, despite the fact that a precut sphincterotomy had been performed. When withdrawing the endoscope, bile was observed to be draining from the minor papilla. The minor papilla was cannulated and the ERCP was successful. Captured images showed the normal accessory pancreatic duct and the distal CBD joining the pancreatic dorsal duct. The patient was transferred to the surgical department and received cholecystectomy and biliary-enteric Roux-en-Y anastomosis. The surgery confirmed the diagnostic accuracy of ERCP. During the next 4 years follow-up, the patient lived non-eventfully.

### Case 3

A 26-year-old female, who had undergone a cholecystectomy and CBD exploration, was admitted having experienced abdominal pain in the right upper quadrant for ~2 months. T-tube imaging showed that the distal CBD of the patient was narrow and tortile so that the contrast agent (Iohexol; YZJ group, Taizhou, China) was unable to discharge into the duodenal lumen. Duodenoscopy revealed a normal structure of the major papilla but it was not possible to perform ERCP due to complex cannulation. Subsequently, the patient received percutaneous choledochoscopy (PTC) and imaging demonstrated that an orifice existed at the distal end of the CBD, but the contrast agent was unable to access the duodenal lumen. In order to determine whether a malignant obstruction was present, ERCP was performed on the patient one month later. The endoscopic procedure was successful when cannulation from the minor papilla was carried out. Following dilation of the distal CBD with a balloon, a contrast agent was injected into CBD and an X-ray revealed a cystic dilation at the distal CBD. Following further dilation of the minor papilla with a balloon, biliary and pancreatic juice was observed to discharge into the duodenal lumen. Finally a plastic pancreatic stent was placed for drainage. Subsequently, stent replacement was conducted every 3–6 months and no further procedural complications were encountered.

### Case 4

**Figure 4:**
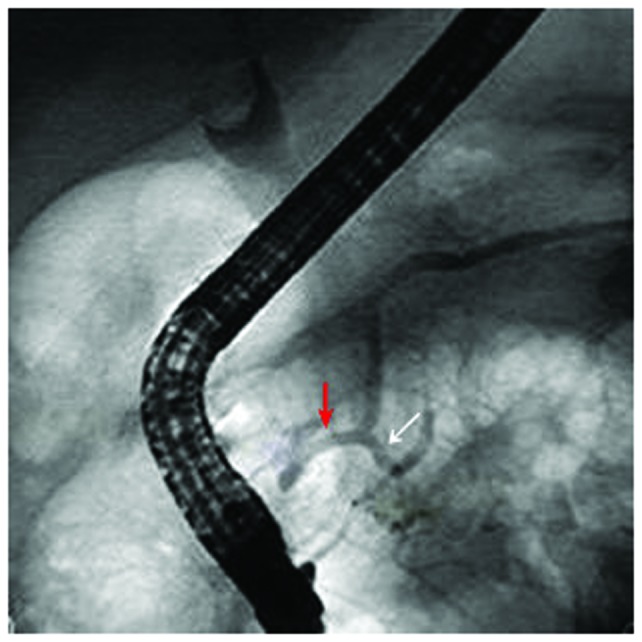
Case 4. Fluoroscopy image shows the anomaly including the pancreaticobiliary maljunction (white arrow) and pancreas divisum (red arrow).

A 70-year-old female presented with a three year history of recurrent episodes of abdominal pain in the right upper quadrant and two months of exacerbation. The patient’s amylase levels were normal. However, B-mode ultrasound had revealed cholelithiasis; therefore, the patient received a cholecystectomy two months prior to presentation. However, the symptoms reappeared and a second B-mode ultrasound conducted one month prior to presentation revealed right intrahepatic bile duct stones. CT and MRCP revealed that the distal CBD was dilated; therefore, ERCP was performed. Following the failure of cannulation from the major papilla, cannulation from the minor papilla was successfully achieved. An X-ray revealed that the distal CBD had joined the pancreatic dorsal duct and subsequently a plastic biliary stent was placed for drainage (Fig. 4). According to the wishes of the patient’s relatives, the patient was discharged after seven days of ERCP treatment when the abdominal pain had ceased. The patient was unavailable for follow-up.

## Discussion

In patients with PBM, the anatomical maljunction of the pancreatic duct and the biliary duct is located outside of the duodenal wall so that the sphincter of Oddi does not directly affect the junction. As the hydropressure in the pancreatic duct is higher than that in biliary duct, pancreatic juice frequently refluxes into the biliary duct in PBM (7), which usually causes lithogenesis, recurrent pancreatitis or intermittent upper abdominal pain with elevation of amylase levels and liver damage (8). PD is a congenital anomaly in which the dorsal and ventral pancreatic ducts drain separately into the duodenum. The main drainage duct of the dorsal pancreatic bud is the accessory pancreatic duct and, in the embryo, it drains bile into the duodenum from the minor duodenal papilla in the embryo. As the body grows, the duct of the dorsal bud atrophies, losing its drainage function (9). Whether PD causes pancreatitis or other complications remains controversial. In the current study, all four cases exhibited both congenital anomalies.

McMahon *et al* reported an anomalous communication between the dorsal pancreatic duct and the CBD via a small ventral pancreatic duct branch. The patient presented with a distal biliary stricture, but the MRCP did not reveal the anomaly. The disease was confirmed by MRCP with intravenous secretin administration, and the patient received a Whipple pancreaticoduodenectomy (10). Terui *et al* studied 78 children with PBM and PD, and concluded that PD rarely combines with PBM and does not cause pancreatitis due to the fact that the incidence of pancreatitis in PD combined with PBM is commensurate with that of isolated PD (11). However, cases where the CBD joins the dorsal pancreatic duct have rarely been reported.

The majority of PD patients remain asymptomatic throughout their lives. However, once symptoms such as jaundice or upper abdominal pain appear, MRCP is the first choice for diagnosis, rather than ERCP, as it is non-invasive. However, MRCP is incapable of revealing the variant anatomy due to the absence of fluid in the CBD or the pancreatic duct, even when secretin is used (13). Diagnostic accuracy may be increased using three-dimensional or dynamic MRCP with secretin stimulation (12). For patients with anatomical maljunction, ERCP remains the gold standard procedure.

Therapeutic options for a patient suffering from PD combined with PBM include open surgery and ERCP, for removal of the stones or the implantation of a stent across the stricture. However, patients are likely to be confronted with the failure of cannulation during ERCP therapy. With dilating CBD images and difficult cannulation during ERCP, many endoscopists tend to select precut sphincterectom. However, the lack of a patient’s medical history, inexperience of the endoscopist, repeated cannulation and precut sphincterotomy may increase the risk of perforation, bleeding and other complications. The existence of an anatomical maljunction is also a risk factor of failure for a precut sphincterectomy, and requires caution. To date, there are no guidelines showing the exact indications for precut sphincterotomy or cannulation of the minor papilla when performing ERCP. Kouchi *et al* support cannulation of the minor papilla when the main pancreatic duct is undetected following cannulation of the major papilla alone, in children with choledochal cysts (14).

Considering the anomalous fusion position of the CBD and the pancreatic duct, a tentative minor papilla cannulation is recommended when repeated cannulation to the major papilla fails for patients with distal biliary stricture, even if the patient’s MRCP does not reveal a pancreatobiliary anomaly.
